# Associations between serum mineral concentrations and mortality by renal function in the Ludwigshafen Risk and Cardiovascular Health Study

**DOI:** 10.1038/s41598-024-79575-w

**Published:** 2024-11-19

**Authors:** Angela P. Moissl, Graciela E. Delgado, Marcus E. Kleber, Bernhard K. Krämer, Winfried März, Stefan Lorkowski

**Affiliations:** 1 Institute of Nutritional Sciences, Friedrich Schiller University Jena, Dornburger Straße 25, 07743 Jena, Germany; 2 Competence Cluster for Nutrition and Cardiovascular Health (nutriCARD) Halle-Jena-Leipzig, Jena, Germany; 3 Vth Department of Medicine (Nephrology, Hypertensiology, Endocrinology, Diabetology, Rheumatology, Pneumology), Medical Faculty Mannheim, University of Heidelberg, Mannheim, Germany; 4 European Center for Angioscience (ECAS), Faculty of Medicine, University of Heidelberg, Mannheim, Germany; 5 Center for Preventive Medicine and Digital Health Baden-Württemberg (CPDBW), Medical Faculty Mannheim, Heidelberg University, Mannheim, Germany; 6 SYNLAB MVZ für Humangenetik Mannheim, Mannheim, Germany; 7 Clinical Institute of Medical and Chemical Laboratory Diagnostics, Medical University of Graz, Graz, Austria; 8 SYNLAB Academy, SYNLAB Holding Deutschland, Augsburg and Mannheim, Mannheim, Germany

**Keywords:** Minerals, Renal function, Cardiometabolic risk, All-cause mortality, Cardiovascular mortality, Biomarkers, Cardiology, Health care, Medical research, Nephrology, Risk factors

## Abstract

The association of serum concentrations of minerals and phosphate with overall and cardiovascular mortality based on renal function is poorly understood. 3307 patients (average age 62.7 ± 10.6 years) in the Ludwigshafen Risk and Cardiovascular Health (LURIC) study were grouped by estimated glomerular filtration rate (eGFR) into three categories: < 60, 60–89, and ≥ 90 mL/min per 1.73 m^2^, per KDIGO 2022 guidelines and were analysed using Cox regression. Low serum sodium and iron concentrations were associated with poor renal function and increased overall mortality risk, whereas higher serum zinc concentrations were associated with reduced overall and cardiovascular mortality risk. Elevated serum copper concentrations were associated with increased mortality risk across all eGFR categories. Comparing low and normal eGFR, we observed a fourfold increase in all-cause mortality risk for eGFR < 60 mL/min per 1.73 m^2^ and a twofold increase for eGFR 60–89 mL/min per 1.73 m^2^, accompanied by changes in serum mineral concentrations. The optimal range of mineral and phosphate concentrations in serum was strongly related to renal function. To reduce mortality risk, it’s important to regularly monitor serum mineral and phosphate concentrations as well as renal function, especially in cardiovascular patients with compromised renal function.

**Supplementary Information:**

The online version contains supplementary material available at 10.1038/s41598-024-79575-w.

## Introduction

Some minerals are essential micronutrients for human health^1,2^. Good micronutrient sources are fruits and vegetables, but meat and fish are also available for some micronutrients. Micronutrients in food usually ensure good bioavailability but must be ingested in sufficient quantities^3^. Since serum concentrations of micronutrients are low, avoiding insufficient micronutrient intake is even more critical, as their deficiency negatively affects human health^4–6^. About two billion people worldwide are affected by micronutrient deficiencies^2,3^. Micronutrient deficiency is most common in children up to five years of age due to malnutrition^7^, pregnant women and older adults^8^. Higher concentrations of micronutrients may be required in some physiological and pathophysiological conditions^1^. If oral intake does not sufficiently meet this requirement, there is an increased risk of developing a chronic nutrient deficiency^9^. Multiple micronutrient deficiencies often occur at the same time^2^. One of the most crucial electrolytes in the body is sodium, which, due to its osmotic activity, plays an essential role in regulating water balance^1^. Together with potassium, it has a crucial role in the nervous system and is required for muscle contraction, heart function, and blood pressure regulation^2^. Magnesium is essential for energy metabolism, muscle contraction, and nerve function^3^. Calcium is crucial for bone health, blood clotting, muscle contraction, and signalling between cells in the body^4^. Iron, a vital trace element, is essential for oxygen transport in the blood and cell metabolism^2,5^. Zinc plays a crucial role in immune function, cell division, and wound healing^2^, while copper acts as a cofactor for enzymes involved in iron metabolism and pigmentation^6^. Inorganic phosphate is crucial for bone health, energy metabolism, and regulating enzyme activities in the body. When renal function is impaired, elevated serum concentrations of electrolytes, such as potassium, magnesium, and inorganic phosphate, may occur, leading to an imbalance in electrolyte homeostasis^3,10^. Calcium, magnesium, iron, copper, zinc, and inorganic phosphate are absorbed from dietary sources in the gastrointestinal tract and are excreted by the kidneys^1,11^. If absorption of these nutrients is impaired or food intake is inadequate, nervous system stimulus transmission^12^, immune response^13^, metabolic processes^14^, haematopoiesis^15^ and protein synthesis^16^ may be impaired. Some studies revealed associations between intake of minerals and cardiovascular disease^17,18^. Dialysis patients or patients with chronic kidney disease (CKD) are often screened for cardiovascular diseases (CVD), as even mild dysfunction increases the risk of CVD^22,23^. Many studies have identified age as an independent risk marker for cardiovascular morbidity^26^, and the physiological function of the kidney declines with increasing age^7^. Thus, the estimated glomerular filtration rate (eGFR) has been established as an independent risk marker^8^.

Since the contribution of minerals to overall and cardiovascular mortality is still discussed^9,10^, we examined the association of serum concentrations of selected minerals and phosphate with mortality stratified by renal function in a patient population at moderate to high cardiovascular risk.

## Results

We investigated the relationship between serum concentrations of selected minerals and inorganic phosphate with all-cause and cardiovascular mortality depending on renal function in 3307 individuals of the Ludwigshafen Risk and Cardiovascular Health (LURIC) cohort. We confirm the decline in renal function with advancing age within our cohort. Patients with poorer renal function were more often female and notably older (*p* < 0.001). Although differences in body mass index (BMI) were small, they were statistically significant (*p* < 0.001) (see Table 1).

**Table 1:** Study characteristics according to eGFR categories.

Variable	Total	Poor renal function	Satisfactory renal function	Good renal function		
eGFR categories		eGFR < 60	eGFR 60–89	eGFR ≥ 90	p^a^	FDR^b^
N	3.307	456	1.642	1.209	–	–
eGFR (mL/min per 1.73 m^2^)	81.7 ± 20.1	46.2 ± 11.5	77.1 ± 8.5	101.5 ± 8.0	< 0.001	< 0.001
Age (years)	62.7 ± 10.6	70.8 ± 8.4	65.5 ± 8.4	55.8 ± 10.2	< 0.001	< 0.001
Female sex (%)	30.3	41.9	33.5	21.8	< 0.001	< 0.001
Body mass index (kg/m^2^)	27.5 ± 4.1	27.5 ± 4.4	27.7 ± 4.0	27.2 ± 4.0	0.020	0.023
LDL-cholesterol (mg/dL)	116.6 ± 34.1	111.0 ± 34.2	117.0 ± 33.6	117.0 ± 35.2	0.002	0.003
HDL-cholesterol (mg/dL)	38.7 ± 10.8	36.8 ± 11.0	39.0 ± 10.8	39.1 ± 10.7	< 0.001	< 0.001
Apolipoprotein A1 (mg/dL)	129.0 ± 25.0	125.0 ± 25.9	130.0 ± 24.7	130.0 ± 24.9	< 0.001	< 0.001
Apolipoprotein A2 (mg/dL)	41.6 ± 9.5	37.4 ± 8.9	41.0 ± 9.1	43.9 ± 9.5	< 0.001	< 0.001
Triglycerides (mg/dL)	147 (109–201)	159 (119–209)	145 (107–198)	144 (108–200)	0.005	0.006
Fasting glucose (mg/dL)	102.4 (93.7–118.5)	105.0 (95.4–125.0)	103.0 (94.3–119)	101.0 (92.7–114.0)	< 0.001	< 0.001
HbA1c (%)	6.3 ± 1.3	6.7 ± 1.4	6.4 ± 1.2	6.1 ± 1.2	< 0.001	< 0.001
Systolic blood pressure (mmHg)	141.1 ± 23.6	145.0 ± 25.5	143.0 ± 23.8	136.0 ± 21.8	< 0.001	< 0.001
Diastolic blood pressure (mmHg)	81.0 ± 11.5	79.2 ± 11.9	81.8 ± 11.6	80.5 ± 10.9	< 0.001	< 0.001
Vitamin D 0.25 (OH) (nmol/L)	17.4 ± 9.7	14.5 ± 9.1	17.7 ± 10.1	18.1 ± 9.2	< 0.001	< 0.001
Vitamin D 1.25 (OH) (nmol/L)	35.0 ± 13.9	24.8 ± 11.0	35.2 ± 13.3	38.5 ± 14.0	< 0.001	< 0.001
High-sensitive C-reactive protein (mg/L)	3.4 (1.3–8.6)	6.5 (2.5–14.7)	3.5 (1.4–8.7)	2.4 (1.0–6.7)	< 0.001	< 0.001
NT-pro-BNP (pg/mL)	293 (106–869)	1180 (482–2740)	332 (140–953)	146 (63–391)	< 0.001	< 0.001
Galectin-3 (ng/mL)	14.5 (11.4–18.4)	22.2 (17.8–27.4)	15.2 (11.9–19.1)	12.4 (9.2–15.3)	< 0.001	< 0.001
Creatinine (mg/dL)	0.9 (0.8–1.0)	1.3 (1.1–1.5)	1.0 (0.9–1.1)	0.8 (0.8–0.9)	< 0.001	< 0.001
Renin (pg/mL)	19.0 (10.0–41.0)	33.0 (14.0–83.8)	18.0 (9.0–40.0)	18.0 (10.0–34.0)	< 0.001	< 0.001
Blood urea nitrogen (mg/dL)	39.5 ± 15.6	60.6 ± 24.6	38.7 ± 9.8	32.4 ± 8.2	< 0.001	< 0.001
Uric acid (mg/dL)	4.9 (4.0–6.0)	6.1 (4.9–7.6)	4.9 (4.0–6.0)	4.4 (3.7–5.3)	< 0.001	< 0.001
Trimethyl-N- aminoxide (µmol/L)	4.24 (2.97–6.12)	6.86 (4.94–10.8)	4.39 (3.30–6.11)	3.31 (2.38–4.76)	< 0.001	< 0.001
Symmetric dimethylamine (µmol/L)	0.54 (0.46–0.65)	0.82 (0.66–1.02)	0.56 (0.48–0.65)	0.47 (0.41–0.54)	< 0.001	< 0.001
Coronary artery disease > 20 (%)	78	83.8	79.9	72.9	< 0.001	< 0.001
Coronary artery disease > 50 (%)	68.3	74.8	69.4	64.4	< 0.001	< 0.001
Hypertension (%)	59	84	77.6	61.9	< 0.001	< 0.001
Hypertension medication (%)	86.8	95.6	88.8	80.7	< 0.001	< 0.001
Diabetes mellitus (%)	40	56.4	42.4	30.4	< 0.001	< 0.001
Smoking status						
Active/former/never (%)	23.4 / 41.1 / 35.5	13.4 / 44.3 / 42.3	19.4 / 42.0 / 38.7	32.5 / 38.7 / 28.8	< 0.001	< 0.001
Cigarette packs per day (%)	10.0 (0.0–30.0)	16.9 (5.0–32.2)	15.7 (5.0–35.0)	20.0 (6.7–37.5)	0.003	0.004
Alcohol consumption (g ethanol /day)	16.0 ± 23.7	10.5 ± 17.0	16.3 ± 23.1	17.6 ± 26.3	< 0.001	< 0.001

Values are given as either median (25th and 75th percentile) for non-normally distributed data, mean ± SD for normally distributed data, or.

percentage for categorical data.

^a^ ANOVA (non-normally distributed variables were log-transformed before entering analyses) or χ^2^ test.

^b^p-Value after FDR correction.

All tests were two-sided; a p-value < 0.05 was considered statistically significant.

eGFR, estimated glomerular filtration rate (mL/min/1.73 m²); FDR, false detection rate; HbA1c, Glycated haemoglobin A1c; HDL, high-density lipoprotein; LDL, low-density lipoprotein; NT-pro-BNP, N-terminal pro-B- type natriuretic peptide-1.

We then examined disparities in cardiovascular risk factors among the eGFR categories (see Table 1). Concentrations of low-density lipoprotein cholesterol (LDL-C; *p* = 0.002) and high-density lipoprotein cholesterol (HDL-C; *p* < 0.001) were notably lower in the eGFR < 60 mL/min per 1.73 m^2^ group compared to the eGFR ≥ 90 mL/min per 1.73 m^2^ category. Similar trends were observed for apolipoprotein-A1 (apoA1; *p* < 0.001), apolipoprotein-A2 (apoA2; *p* < 0.001), and diastolic blood pressure (*p* < 0.001). Conversely, plasma triglycerides, fasting glucose, glycated haemoglobin A_1c_ (HbA1c), high-sensitive C-reactive protein (hs-CRP), N-terminal pro-B-type natriuretic peptide-1 (NT-pro-BNP) and systolic blood pressure were significantly higher (*p* < 0.001) in patients with impaired renal function.

To characterise renal (dys-)function within our cohort, we analysed renal-associated markers, including galectin-3, creatinine, renin, urea, uric acid, trimethyl-N-aminoxide (TMAO), and symmetric dimethylamine (SDMA). All these markers were significantly elevated (*p* < 0.001) in the poor renal function group compared to the good renal function group. In the overall cohort, serum concentrations of Vitamin D 0.25 (OH) (nmol/L) were 17.4 ± 9.7, with the lowest concentration observed in the eGFR category < 60 (14.5 ± 9.1) and the highest concentrations in the eGFR category > 90 (18.1 ± 9.2, *p* < 0.001). Similarly, for Vitamin D 1.25 (OH) (nmol/L), the lowest concentration was found in the eGFR category < 60 (24.8 ± 11.0), in contrast to intermediate levels in the eGFR 60–89 category, and the highest concentration in the eGFR > 90 category (38.5 ± 14.0, *p* < 0.001).

We subsequently investigated whether there were differences in the incidence of CVD among the three eGFR categories. Indeed, patients with poor renal function (eGFR < 60 mL/min per 1.73 m^2^) more frequently presented with coronary artery disease (CAD), hypertension, and diabetes mellitus (*p* < 0.001). Conversely, alcohol consumption and cigarette smoking were more prevalent in individuals with good renal function than in those with poor renal function.

### Differences in serum concentrations of minerals and phosphate depending on renal function

Next, we examined the differences in serum concentrations of minerals and inorganic phosphate across the three eGFR categories (see Table 2). No physiologically relevant differences were found in serum concentrations of sodium and calcium between the groups. Potassium, magnesium, copper, and inorganic phosphate were higher in the low eGFR category compared to the high eGFR category. In contrast, serum concentrations of iron and zinc were significantly lower (*p* < 0.001) in the low eGFR category compared to the other two categories. Supplementary Table S1 shows the p-values for groupwise comparisons of serum mineral and phosphate concentrations between the eGFR categories.

**Table 2:** Micronutrients according to eGFR categories.

Variable	Total	Poor renal function	Satisfactory renal function	Good renal function	p^a^	FDR^b^
eGFR categories		eGFR < 60	eGFR 60–89	eGFR ≥ 90		
N	3.307	456	1.642	1.209		
Sodium (mmol/L)	141 (139–143)	141 (139–143)	141 (140–143)	141 (139–143)	0.002	0.003
Potassium (mmol/L)	4.2 (4.0–4.4)	4.3 (4.0–4.5)	4.2 (4.0–4.4)	4.2 (4.0–4.4)	< 0.001	< 0.001
Magnesium (mmol/L)	0.85 (0.79–0.91)	0.88 (0.82–0.95)	0.85 (0.79–0.91)	0.84 (0.78–0.90)	< 0.001	< 0.001
Calcium (mmol/L)	2.33 (2.26–2.40)	2.33 (2.26–2.42)	2.33 (2.26–2.39)	2.32 (2.27–2.39)	< 0.001	0,699
Iron (µg/dL)	89 (66–114)	75 (57–100)	89 (66–114)	93 (72–119)	< 0.001	< 0.001
Zinc (µmol/L)	86 (77–96)	81 (70–90)	86 (77–95)	88 (79–98)	< 0.001	< 0.001
Copper (µg/dL)	105 (91–122)	113 (99–130)	106 (91–122)	103 (90–120)	< 0.001	< 0.001
Inorganic phosphate (mg/dL)	3.5 (3.1–3.9)	3.6 (3.2–4.1)	3.5 (3.1–3.9)	3.5 (3.1–3.8)	< 0.001	< 0.001

Values are given as either median (25th and 75th percentile) for non-normally distributed data as mean ± SD for normal distributed data or percentage for categorial data.

^a^ An ANOVA (non-normally distributed variables were log-transformed before entering analyses) or χ2 test.

^b^p-Value after FDR correction.

All tests were two-sided; a p-value < 0.05 was considered statistically significant.

eGFR, estimated glomerular filtration rate (mL/min/1.73 m²); FDR, false detection rate.

### Association of serum concentrations of minerals and phosphate with all-cause mortality and cardiovascular mortality

**Fig. 1:**
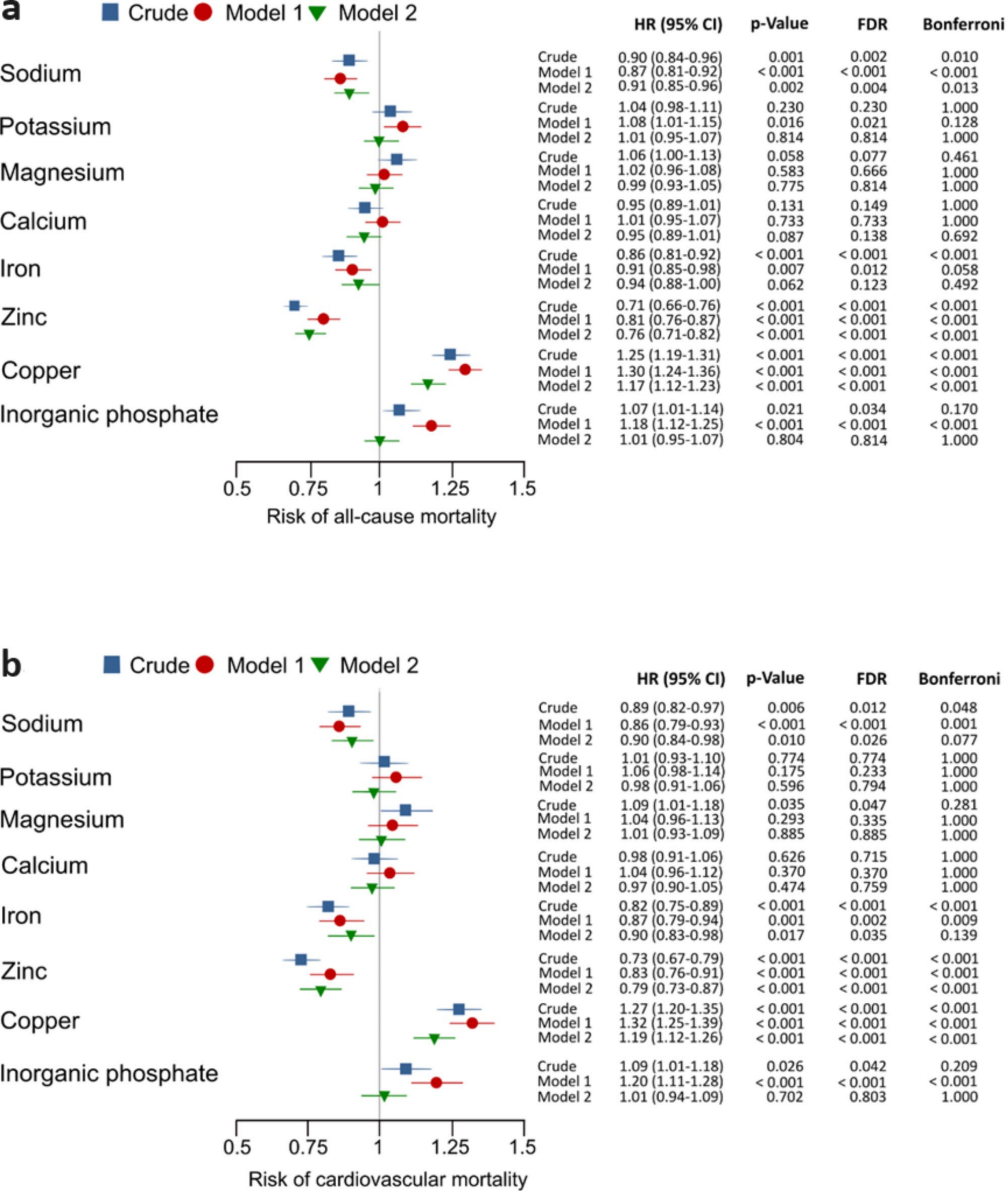
All-cause and cardiovascular mortality risk. Risk for **(a)** all-cause and **(b)** cardiovascular mortality. ***Crude***: unadjusted. ***Model 1***: adjusted for age, gender, and BMI. ***Model 2***: adjusted only for estimated glomerular filtration rate. HR, hazard ratios (95% confidence intervals); FDR, false discovery rate; Bonferroni, statistical significance after Bonferroni correction for multiple testing.

Associations of the different minerals and inorganic phosphate with the risk of all-cause and cardiovascular mortality were examined using Cox regression models (see Fig. 1). Consistent direct associations were found for copper and inorganic phosphate, while inverse associations were observed for sodium, iron, and zinc, both in the crude Model and in Model 1, adjusted for age and sex. Adjustment for eGFR only (Model 2) abolished the association of inorganic phosphate and attenuated the association of calcium with mortality but had negligible effects on the relationships of the other minerals with mortality risk. Following these preliminary analyses, we further examined Model 2 with eGFR adjustment in more detail. We investigated the association with mortality across three eGFR categories (< 60, 60–89, and ≥ 90 mL/min/1.73 m²). The eGFR stratification revealed an interaction between renal function and serum concentrations of minerals and inorganic phosphate.

Therefore, we analysed how renal function affects the associations of serum concentrations of minerals and inorganic phosphate with mortality risk. Higher serum sodium concentrations were associated with a lower risk for all-cause mortality in the intermediate [HR (95% CI)] of [0.88 (0.81–0.96), *p* = 0.005] and high [0.85 (0.74–0.97), *p* = 0.019] eGFR category. Higher serum concentrations of calcium [0.92 (0.84–1.00), *p* = 0.055] and iron [0.89 (0.81–0.97), *p* = 0.009] were associated with a reduced risk of all-cause mortality only in the middle eGFR category. In comparison, higher serum concentrations of zinc were associated with a reduced risk of all-cause mortality in all three renal function categories (eGFR < 60 mL/min/1.73 m^2^, [0.78 (0.69–0.89), *p* < 0.001], eGFR 60–89 mL/min/1.73 m^2^, [0.75 (0.68–0.83), *p* < 0.001], eGFR ≥ 90 mL/min/1.73 m^2^ [0.79 (0.69–0.91), *p* = 0.001]). In contrast, higher serum magnesium concentrations were associated with an increased risk of all-cause mortality in the low eGFR category [1.13 (1.01–1.27), *p* = 0.033]. For higher serum copper concentrations, we found an increased risk of all-cause mortality in all three renal function categories (eGFR < 60 mL/min/1.73 m²; [1.12 (1.01–1.23), *p* = 0.031], eGFR 60–89 mL/min/1.73 m^2^; [1.20 (1.11–1.29), *p* < 0.001], eGFR ≥ 90 mL/min/1.73 m^2^; [1.29 (1.15–1.45), *p* < 0.001]). No significant association was observed for serum concentrations of potassium and inorganic phosphate.

We found similar associations with cardiovascular mortality for higher serum concentrations of sodium, magnesium, and copper. In contrast to overall mortality, we observed no associations between serum concentrations of calcium and iron with cardiovascular mortality risk. Higher serum zinc concentrations were associated with a reduced risk of cardiovascular mortality in the category with low renal function [eGFR < 60 mL/min/1.73 m^2^; 0.83 (0.71–0.96), *p* = 0.016] and intermediate renal function category [eGFR 60–89 mL/min/1.73 m^2^; 0.76 (0.67–0.86), *p* < 0.001], but no association was observed in the category with best renal function [eGFR ≥ 90 mL/min/1.73 m^2^; 0.87 (0.72–1.04), *p* = 0.125]. As with all-cause mortality, serum concentrations of potassium and inorganic phosphate showed no association with cardiovascular mortality risk (see Supplementary Fig. S1).

### Interactions of serum minerals and renal function

We analysed six different models to investigate potential interactions between serum concentrations of minerals and phosphate with kidney function regarding the risk of all-cause mortality. We found that all serum concentrations of minerals and phosphate were associated with a fourfold higher risk of all-cause mortality in the low eGFR category, while the satisfactory eGFR category was associated with a twofold higher risk than the normal renal function category. We obtained significance for all interactions (*p* < 0.001), as detailed in Table 3. Similar estimates were observed for cardiovascular mortality, and these results are presented in Supplementary Table S2.

**Table 3:** Cox regression models for all-cause mortality including interaction analysis of micronutrients with eGFR categories (HR (95% CI).

Mineral	< 60 eGFR	60–89 eGFR	≥ 90 eGFR	Interaction *p*-value	eGFR whole cohort	*p*-Value
Sodium	–	-	–	–	0.97 (0.97–0.98)	< 0.001
Model 1	0.96 (0.78–1.17)	1.09 (0.95–1.24)	1.00 (ref)	–	–	–
Model 2	0.98 (0.86–1.10)	0.88 (0.81–0.96)	1.00 (ref)	–	–	–
Model 3	4.91 (4.09–5.88)	2.03 (1.73–2.39)	1.00 (ref)	–	–	–
Model 4	–	–	–	< 0.001	–	–
Model 5	–	–	–	< 0.001	–	–
Model 6	–	–	–	< 0.001	–	–
Potassium				–	1.01 (0.95–1.07)	0.814
Model 1	0.96 (0.78–1.17)	1.09 (0.95–1.24)	1.00 (ref)	–	–	–
Model 2	1.06 (0.94–1.19)	0.97 (0.89–1.06)	1.00 (ref)	–	–	–
Model 3	4.92 (4.10–5.90)	2.01 (1.71–2.36)	1.00 (ref)	–	–	–
Model 4	–	–	–	< 0.001	–	–
Model 5	–	–	–	< 0.001	–	–
Model 6	–	–	–	< 0.001	–	–
Magnesium				–	0.99 (0.93–1.05)	0.775
Model 1	0.96 (0.78–1.17)	1.09 (0.95–1.24)	1.00 (ref)	–	–	–
Model 2	1.13 (1.01–1.27)	0.93 (0.85–1.01)	1.00 (ref)	–	–	–
Model 3	4.95 (4.12–5.94)	2.01 (1.71–2.36)	1.00 (ref)	–	–	–
Model 4	–	–	–	< 0.001	–	–
Model 5	–	–	–	< 0.001	–	–
Model 6	–	–	–	< 0.001	–	–
Calcium				–	0.95 (0.89–1.01)	0.087
Model 1	0.96 (0.78–1.17)	1.09 (0.95–1.24)	1.00 (ref)	–	–	
Model 2	0.95 (0.84–1.07)	0.92 (0.84–1.00)	1.00 (ref)	–	–	–
Model 3	4.94 (4.12–5.92)	2.01 (1.71–2.37)	1.00 (ref)	–	–	–
Model 4	–	–	–	< 0.001	–	–
Model 5	–	–	–	< 0.001	–	–
Model 6	–	–	–	< 0.001	–	–
Iron				–	0.94 (0.88–1.00)	0.062
Model 1	0.96 (0.78–1.17)	1.09 (0.95–1.24)	1.00 (ref)	–	–	–
Model 2	1.00 (0.88–1.13)	0.89 (0.81–0.97)	1.00 (ref)	–	–	–
Model 3	4.78 (3.98–5.74)	1.99 (1.70–2.34)	1.00 (ref)	–	–	–
Model 4	–	–	–	< 0.001	–	–
Model 5	–	–	–	< 0.001	–	–
Model 6	–	–	–	< 0.001	–	–
Zinc					0.76 (0.71–0.82)	< 0.001
Model 1	0.96 (0.78–1.17)	1.09 (0.95–1.24)	1.00 (ref)	–	–	–
Model 2	0.78 (0.69–0.89)	0.75 (0.68–0.83)	1.00 (ref)	–	–	–
Model 3	4.43 (3.89–5.31)	1.95 (1.66–2.29)	1.00 (ref)	–	–	–
Model 4	–	–	–	< 0.001	–	–
Model 5	–	–	–	< 0.001	–	–
Model 6	–	–	–	< 0.001	–	–
Copper					1.17 (1.12–1.23)	< 0.001
Model 1	0.96 (0.78–1.17)	1.09 (0.95–1.24)	1.00 (ref)	–	–	–
Model 2	1.12 (1.01–1.23)	1.20 (1.11–1.29)	1.00 (ref)	–	–	–
Model 3	4.58 (3.82–5.51)	1.97 (1.68–2.32)	1.00 (ref)	–	–	–
Model 4	–	–	–	< 0.001	–	–
Model 5	–	–	–	0.009	–	–
Model 6	–	–	–	< 0.001	–	–
Inorganic phosphate					1.01 (0.94–1.09)	0.702
Model 1	0.96 (0.78–1.17)	1.09 (0.95–1.24)	1.00 (ref)	–	–	–
Model 2	0.99 (0.88–1.12)	0.99 (0.91–1.08)	1.00 (ref)	–	–	–
Model 3	4.92 (4.10–5.91)	2.02 (1.72–2.37)	1.00 (ref)	–	–	–
Model 4	–	–	–	< 0.001	–	–
Model 5	–	–	–	< 0.001	–	–
Model 6	–	–	–	< 0.001	–	–

**Model 1**: eGFR continuous; **Model 2**: Mineral continuous; **Model 3**: Mineral continuous + eGFR continuous;

**Model 4**: Mineral + interaction; **Model 5**: eGFR continuous + interaction; **Model 6**: Mineral continuous + eGFR continuous + interaction.

eGFR, estimated glomerular filtration rate; HR, Hazard ratio; ref; reference category.

All tests were two-sided; a p-value < 0.05 was considered statistically significant.

### Hazard ratios of mortality according to renal function

**Fig. 2:**
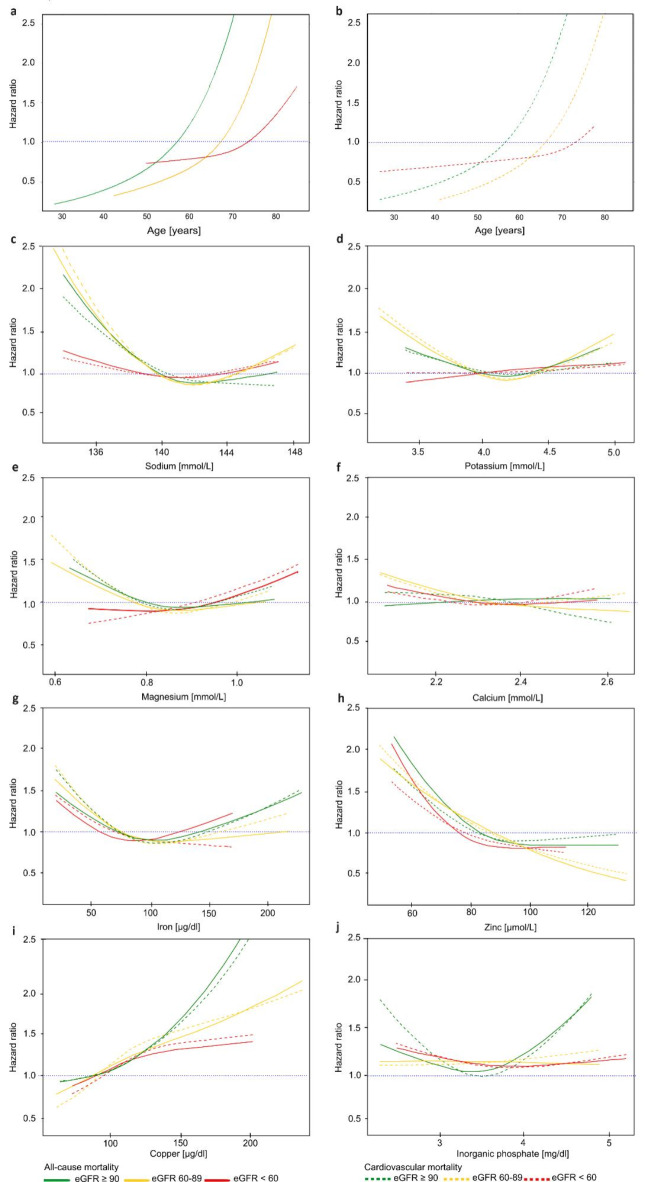
Hazard ratios of micronutrient concentrations and mortality according to three eGFR categories. Serum concentrations were modelled as restricted cubic splines in Cox regression analysis without adjustment and plotted against the hazard ratios. All-cause mortality, solid lines; cardiovascular mortality, dashed lines. ***Color scheme***: green, eGFR ≥ 90 mL/min/1.73m^2^; yellow, eGFR 60–89 mL/min/1.73m^2^; red, eGFR < 60 mL/min/1.73m^2^.

To examine the association of serum concentrations of minerals and inorganic phosphate with the risk of all-cause and cardiovascular mortality, we plotted the hazard ratios of each individual mineral and inorganic phosphate for all-cause and cardiovascular mortality across each renal function category (Fig. 2). As age is a primary determinant of mortality and CVD, we depicted the correlation between age and all-cause cardiovascular mortality within each renal function category in Fig. 2a and 2b. Our analyses indicate a heightened mortality risk with advancing age, irrespective of renal function, as the risk of both all-cause and cardiovascular mortality steadily rises across all three renal function categories.

Figure 2c, 2g and 2j show that all hazard ratio curves exhibit a J- or U-shaped pattern. The concentration dependences highlight optimal serum concentrations for sodium, potassium, magnesium, calcium, iron, and inorganic phosphate as a function of eGFR category and outcome. These concentration ranges were determined by the intersections of each hazard ratio curve with a hazard ratio of 1. The optimal obtained concentration ranges are shown in detail in Supplementary Table S3. For zinc, the risk of all-cause and cardiovascular mortality decreased with rising serum concentration in each eGFR category (Fig. 2h). Notably, with good renal function and increasing serum zinc concentrations, the association with cardiovascular mortality became detrimental. In contrast to zinc, serum copper concentrations exhibited adverse associations with all-cause and cardiovascular mortality above a certain threshold, irrespective of renal function (Fig. 2i).

The risk for all-cause and cardiovascular mortality increased for all minerals and inorganic phosphate, outside optimal serum concentration ranges as shown in Supplementary Table S3.

We conducted sensitivity analyses to identify potential disparities between patients with and without diabetes mellitus. Our findings revealed similar associations in both groups, with no significant deviations from the above results (Supplementary Fig. S2 and Tables S4 to S7).

## Discussion

We examined the associations of serum concentrations of selected minerals and inorganic phosphate with all-cause and cardiovascular mortality. We found that eGFR decreases with age, as does the body’s ability to reabsorb essential minerals^11,12^. This finding is consistent with the results of a review by Noronah et al.^13^, which highlights the decline in glomerular filtration rate as a key factor in the loss of kidney function with increased age. This review also highlights the importance of these age-related changes for the evaluation and management of kidney health in older populations when electrolyte balance is compromised, the kidneys are usually able to compensate^1^. Still, electrolyte balance can become disturbed, especially in the elderly, if mineral concentrations and water content are disrupted, for example, by surgery, severe infection, infusions, or inadequate water and nutrient intake^1,14^.

Previous studies, such as Irsik et al.^15^, have shown that aging significantly impacts mineral homeostasis, particularly in the kidney, leading to reduced calcium and phosphate absorption and contributing to mineral imbalances as kidney function declines. The lower incidence of smoking and reduced alcohol consumption in patients with poor renal function compared with those with good renal function is likely due to both medical advice and interventions.

Elevated serum sodium concentrations in individuals with impaired renal function were associated with an increased overall mortality risk. This is consistent with findings from Liu et al.^16^, who also reported that elevated sodium levels contribute to mortality, particularly in elderly populations with high sodium intake. The study highlighted a non-linear relationship between sodium intake and all-cause mortality, with inflection points that indicate critical thresholds for sodium consumption in relation to health outcomes. This may be explained by impaired sodium regulation when renal function is reduced, affecting vital body functions^17^. Serum iron concentrations and renal function were associated with an increased risk of all-cause mortality depending on renal function status and serum iron concentration. This is consistent with findings from Cheng et al.^18^, which showed that both lower and higher serum ferritin levels were independent risk factors for kidney disease progression and mortality in patients with chronic kidney disease (CKD). Adequate iron is necessary, and its supplementation should be tailored based on the patient’s kidney health to avoid associated risks with mortality^19^.

Our study found that higher serum zinc concentrations above 75.71 µmol/L were associated with reduced overall mortality risks, while serum concentrations exceeding 77.78 µmol/L were associated with decreased cardiovascular mortality risk. Our studies are in line with previous studies, as reported by Knez et al.^20^, which reported similar protective effects of zinc on cardiovascular health. The immunological benefits of zinc may help to explain this association^21–23^.

Conversely, serum copper concentrations higher than 102 µg/dL in patients across all renal function categories were associated with an increase in overall and cardiovascular mortality. This may be due to copper itself, which is essential for the organism in small amounts but can have toxic effects at high concentrations. The toxic impact of copper at elevated serum concentrations can lead to oxidative stress and cellular damage^23–26^.

Low serum concentrations indicate an insufficient reabsorption of minerals due to impaired renal function^25,27^. As expected, we found differences in serum micronutrient concentrations that varied according to the different categories of eGFR^27,28^. Interestingly, our findings diverge slightly from findings reported by Cheng et al.^29^, who reported more pronounced changes in micronutrient concentrations among individuals with early-stage renal dysfunction. This discrepancy could be due to differences in cohort characteristics or a different method of measuring of renal function. The study highlighted that higher zinc and selenium concentrations were positively associated with better renal outcomes, suggesting that micronutrient concentrations may play a critical role in renal health. Substances that are not effectively removed due to renal insufficiency can accumulate in the bloodstream and become harmful^28,30^. Consistent with our expectations, we detected increased potassium, magnesium, copper, and inorganic phosphate concentrations among participants with decreased renal function^28,31^. In addition, we noted an association between serum mineral concentrations with the incidence of diabetes mellitus, hypertension, and cardiovascular diseases in our cohort. This is likely due to reduced renal function^17,31,32^.

Our analysis of serum mineral concentrations per renal function category revealed that the optimal range of serum mineral concentrations is strongly associated with renal function. Upon comparing the optimal concentration ranges with published reference values, we observed that all concentration ranges were within the reference values^33^. While our optimal concentration ranges align with established reference values, some studies, such as Xi et al.^34^, have suggested narrower ranges for certain minerals in patients with severe renal impairment. This indicates the potential need for more specific guidelines based on renal function status. However, according to our analyses, renal function should be considered when evaluating serum mineral concentrations for further interventions, such as nutrient supplementation.

## Conclusions

In our study we could show that inadequate serum mineral concentrations, which can be due to inadequate intake or poor renal function, are associated with mortality. Serum mineral concentrations and renal function should be monitored in elderly patients, particularly if they are at increased cardiovascular risk.

### Strengths and limitations

All participants in the LURIC study were of European heritage. Thus, our results may not be transferable to other ethnicities. Only study participants referred for coronary angiography were included in LURIC, so the results cannot be generalised to a healthy population. Next, no urine samples were available, as well as no status of nutritional intake. Also, eGFR was only calculated once at baseline in LURIC, precluding the definition of CKD stages 1 and 2 according to guidelines. Due to the low number of study participants with severely impaired kidney function (*N* = 48 with eGFR < 30 ml/min/1.73m2) we did not define separate groups for CKD stages 3–5. However, the strength of the LURIC cohort is the comprehensive and accurate clinical and metabolic characterisation of the study participants, including the availability of coronary angiograms and a complete 10-year follow-up on mortality.

## Materials and methods

### Subjects

Between 1997 and 2000, a total of 3,316 individuals who underwent coronary angiography were enrolled in this continuously active prospective investigation. The primary clinical reasons for undergoing angiography were chest pain or a positive non-invasive stress test leading to myocardial ischemia. Those with acute medical conditions other than acute coronary syndrome (ACS), chronic illnesses unrelated to cardiovascular health, or a history of cancer within the preceding five years were excluded from the study.

### Follow-up

Throughout a follow-up period spanning a median of 9.9 years, information regarding the vital status of participants enrolled in this study was gathered from local civil registry offices. Mortality data were acquired through the collection of death certificates. The vital status of all study participants was fully ascertainable, although the cause of death for 19 individuals remained unknown. While these participants were included in calculating all-cause mortality, they were not factored into determining specific causes of death. Along the follow-up, 995 individuals (30%) passed away, with 622 (18.8%) succumbing to cardiovascular causes. Cardiovascular mortality encompassed sudden cardiac death (*N* = 259, 7.9%), fatal myocardial infarction (*N* = 106, 3.2%), death resulting from congestive heart failure (*N* = 148, 4.5%), mortality immediately following coronary heart disease (CHD) intervention treatment (*N* = 26, 0.8%), fatal stroke (*N* = 61, 1.8%), and other causes of death attributable to cardiovascular disease (CVD) (*N* = 19, 0.6%).

### Laboratory procedures

Blood samples were collected by venipuncture from all participants at the beginning of the study. Winkelmann et al. have previously documented the specific sampling and assay methodology^35^. Serum calcium levels were assessed using the o-cresol phthalein complexone colorimetric assay (Roche Diagnostics, Mannheim, Germany) conducted on a Hitachi 717 analyser (Hitachi, Mannheim, Germany). Serum copper was measured utilising the 3,5-diBr-PAESA assay (Rolf Grener Biochemica, Flacht, Germany) on a Cobas Mira Plus Roche analyser (Roche Diagnostics). Serum iron concentrations were determined employing a colorimetric ferrozine assay (Roche Diagnostics) on a Hitachi 717 analyser. Serum magnesium levels were measured through a photometric assay on a Cobas Mira Plus system (Roche Diagnostics). Serum potassium and sodium concentrations were determined utilising an ion-selective electrode (ISE diluent, internal ISE reference, and reference electrode solution; Roche Diagnostics) on a Hitachi 717 system. Zinc levels were assessed in serum using a photometric colorimetric assay (5-bromium, PAPS, zinc complex; Wako Chemicals, Neuss, Germany) on a Cobas Mira Plus system (Roche Diagnostics). Inorganic phosphate levels were evaluated in blood serum using the molybdate reaction PHOS (Roche Diagnostics) on a Hitachi 717 analyser.

### Definition of clinical variables and endpoints

Data for mineral analysis were available for 3,307 individuals (mean age 62.7 ± 10.6 years). The presence of visible lumen narrowing (> 20% stenosis) in at least one of 15 coronary segments was used to define coronary artery disease (CAD) according to the American Heart Association classification^36^. According to the 2010 American Diabetes Association guidelines, diabetes mellitus is defined as elevated fasting glucose (≥ 126 mg/dl) and glucose after a 2-hour glucose tolerance test (> 200 mg/dl), elevated glycated haemoglobin (Hb)A1c (≥ 6.5%), and a history of diabetes mellitus^37^. According to the 2018 ESC/ESH guidelines for managing arterial hypertension, hypertension was defined as systolic blood pressure ≥ 140 and diastolic blood pressure ≥ 90 mmHg or a history of hypertension^38^. The estimated glomerular filtration rate (eGFR) was calculated using the Chronic Kidney Disease Epidemiologic Collaboration (CKD-EPI) formula^39^. In addition to established parameters such as age, sex, LDL- and HDL-cholesterol, triglycerides, fasting blood glucose, blood pressure, smoking, and alcohol consumption, several biomarkers such as renal function markers and the minerals sodium, potassium, magnesium, calcium, iron, copper, zinc and inorganic phosphate were examined to characterise the study.

### Statistical analyses

Study participants were divided into three different categories of estimated glomerular filtration rate (eGFR), i.e., < 60, 60–89, and ≥ 90 mL/min/ 1.73 m², according to KDIGO2022 (Kidney Disease Improving Global Outcomes). The cut-off values for the eGFR categories were selected from the literature to define clinical cut-off criteria^40^. Cox regression analyses were performed for minerals and all-cause and cardiovascular mortality in three models: a crude unadjusted Model, a sex- and age-adjusted Model 1, and an eGFR-adjusted Model 2. After preliminary analysis, we performed Cox regression analyses for our study cohort in all three eGFR categories. We calculated hazard ratios for the serum concentrations of each mineral to identify the optimal concentration ranges for the different renal function categories. We then compared these ranges with the typical ranges of mineral concentrations reported in the literature^33^. To investigate the potential modification of the relationship between mineral concentrations and all-cause and cardiovascular mortality by renal function, an interaction term was constructed by multiplying mineral concentration with estimated glomerular filtration rate (eGFR). This methodological approach allows for the assessment of how the effect of mineral concentration on the outcome variable may differ across varying levels of renal function, providing insights into complex interdependencies.

Using sensitivity analysis for diabetic and nondiabetic patients according to the American Diabetes Association classification^37^, we tried to verify possible differences in serum mineral concentrations between these two groups of cardiovascular patients. Continuous data are presented as mean (±) standard deviation (SD) for normally distributed data or as median with 25th and 75th percentiles for non-normally distributed data. Categorical data are expressed as percentages. The statistical significance of differences between tertiles was determined by ANOVA (non-normally distributed variables were log-transformed before entering analyses) for continuous variables or by the χ^2^ test for categorical variables. Tukey’s honestly significant difference test was used for analysis^41^. All analyses were performed using R version 4.2.2^42^. Hazard ratio plots were generated using the R package rms version 6.4–1.43. Restricted cubic splines were calculated using the rcs function of the R package rms^43^. The function automatically sets knots at the 10th, 50th, and 90th percentiles of serum mineral concentrations. All tests were two-sided; a p-value < 0.05 was considered statistically significant. Correction for multiple testing was performed using the false discovery rate (FDR) method or Bonferroni correction.

## Electronic supplementary material

Below is the link to the electronic supplementary material.

Supplementary Material 1

## Data Availability

Due to the statutes of the Ludwigshafen Risk and Cardiovascular Health (LURIC) Study GmbH, which must recognise the Federal Data Protection Act and the consent of the study participants, the data cannot be disclosed to the public.The use of the LURIC study database is governed by the statutes of the non-profit Ludwigshafen Risk and Cardiovascular Health (LURIC) Study GmbH, which is registered under the number HRB 742978 at the district court Mannheim, Germany. According to the organisation’s statutes, data may be made available to researchers upon request and approval; such requests may not be unreasonably denied. This procedure means the data cannot be publicly released without formal consent. It ensures that the rules of good scientific practice are followed and that the people responsible for the study’s design and organisation are named.Interested researchers are invited to address their queries or suggestions to Kai Grunwald (kai.grunwald@weitnauer.net) or the principal investigator of the LURIC study, Winfried März (winfried.maerz@luric-online.de, winfried.maerz@synlab.com). Finally, the authors confirm that they have accessed these data with permission from LURIC and that all other researchers can access the data the same way as the authors.

## Abbreviations

ALT: Alanine aminotransferase
AST: Aspartate aminotransferase
BMI: Body mass index
CAD: Coronary artery disease
CHD: Coronary heart disease
CKD: Chronic kidney disease
CVD: Cardiovascular disease
eGFR: Estimated glomerular filtration rate
HbA1c: Glycated haemoglobin A_1c_
HDL-C: High-density lipoprotein cholesterol
hs-CRP: High-sensitive C-reactive protein
LDL-C: Low-density lipoprotein cholesterol
NT-pro-BNP: N-Terminal pro-B-type natriuretic peptide-1
