# Supplementary material for: Associations between serum mineral concentrations and mortality by renal function in the Ludwigshafen Risk and Cardiovascular Health Study
Source: Sci Rep. 2024 Nov 19;14:28581. doi: 10.1038/s41598-024-79575-w (PMC11577029; doi:10.1038/s41598-024-79575-w)
Supplement: Supplementary file 1 — Supplementary Material 1 [file 41598_2024_79575_MOESM1_ESM.docx]

# **Supplementary material**

**Associations between serum mineral concentrations and mortality by renal function in the Ludwigshafen Risk and Cardiovascular Health Study**

Angela P. Moissl^1,2,3^, Graciela E. Delgado^3^, Marcus E. Kleber^3,6^, Bernhard K. Krämer ^3,4,5^, Winfried März^3,7,8^, Stefan Lorkowski,^1,2^

^1^ Institute of Nutritional Sciences, Friedrich Schiller University Jena, Jena, Germany

^2^ Competence Cluster for Nutrition and Cardiovascular Health (nutriCARD) Halle-Jena-Leipzig, Jena, Germany

^3^ Vth Department of Medicine (Nephrology, Hypertensiology, Endocrinology, Diabetology, Rheumatology, Pneumology), Medical Faculty Mannheim, University of Heidelberg, Mannheim, Germany

^4^ European Center for Angioscience (ECAS), Faculty of Medicine of the University of Heidelberg, Mannheim, Germany

^5^ Center for Preventive Medicine and Digital Health Baden-Württemberg (CPDBW), Medical Faculty Mannheim, Heidelberg University, Mannheim, Germany

^6^ SYNLAB MVZ für Humangenetik Mannheim, Mannheim, Germany

^7^ Clinical Institute of Medical and Chemical Laboratory Diagnostics, Medical University of Graz, Graz, Austria

^8^ SYNLAB Academy, SYNLAB Holding Deutschland, Augsburg and Mannheim, Germany

***Address for correspondence:***

Prof. Dr. Stefan Lorkowski

Institute of Nutritional Sciences

Friedrich Schiller University Jena

Dornburger Straße 25

07743 Jena

Germany

E-Mail: stefan.lorkowski@uni-jena.de

**
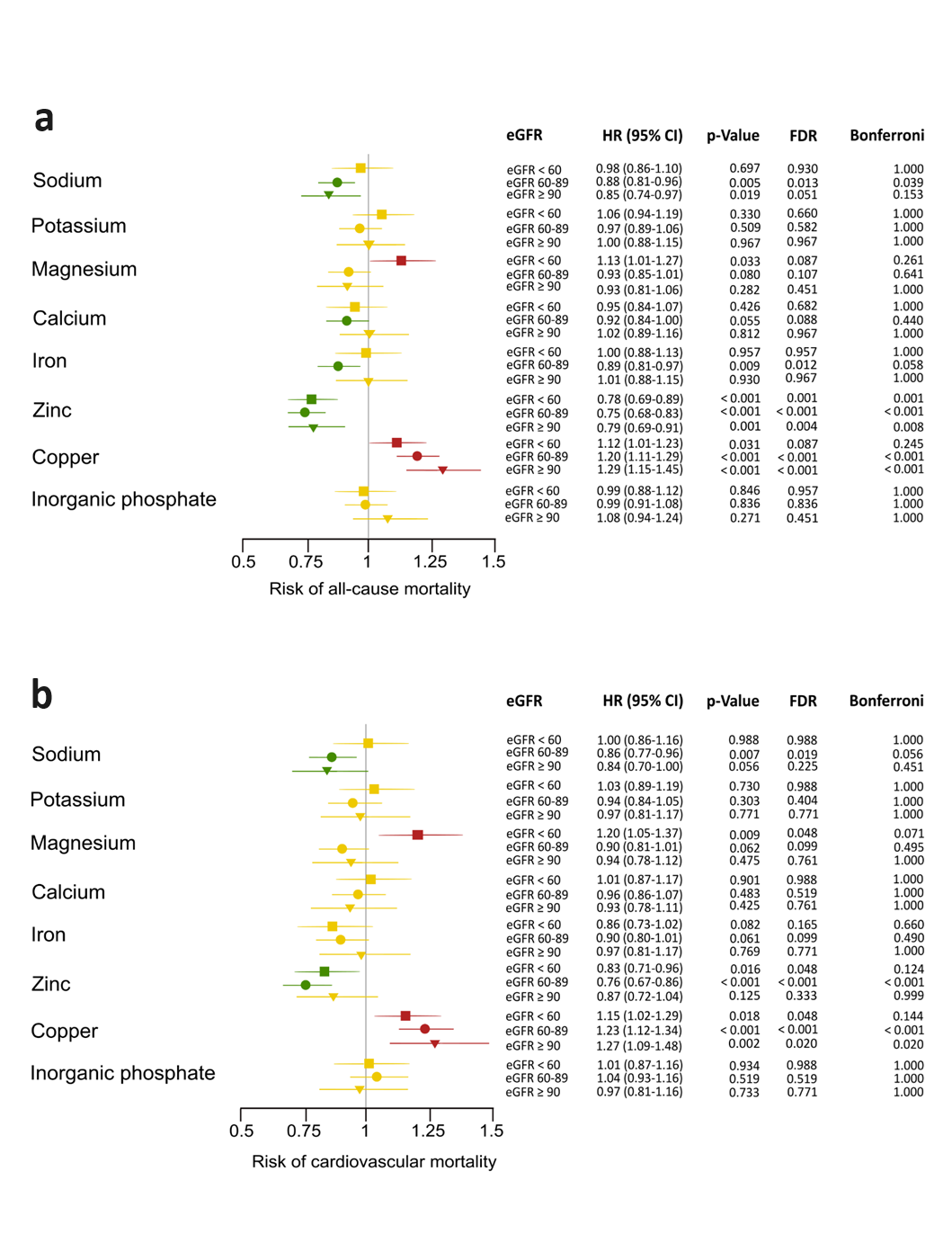
**

**Supplementary Figure S1**: Unadjusted risk stratification depending on renal function categories.

**a)** All-cause mortality and **b)** cardiovascular mortality according to three groups of renal function (squares: eGFR < 60 mL/min/1.73 m^2^; circles: eGFR 60-89 mL/min/1.73 m^2^; triangles: eGFR: ≥ 90 mL/min/1.73 m^2^); green marks a protective, yellow a neutral and red a harmful association with the risk of all-cause and cardiovascular death, respectively. **Abbreviations:** Bonferroni, statistical significance after Bonferroni correction for multiple testing; eGFR, estimated glomerular filtration rate; FDR, false discovery rate; HR, hazard ratios (95% confidence intervals).

All tests were two-sided; a p-value < 0.05 was considered statistically significant.


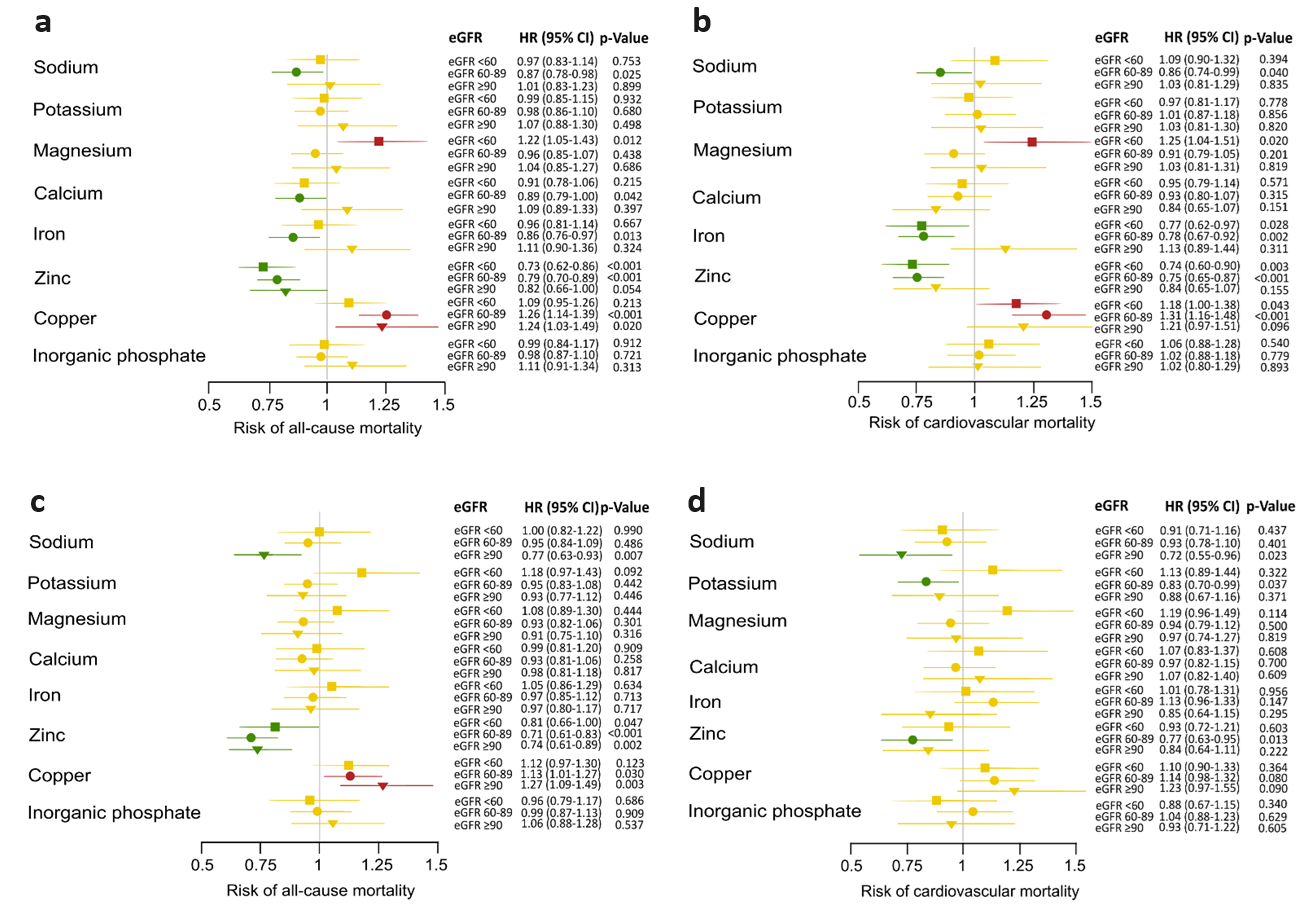


**Supplementary Figure S2: Unadjusted risk stratification depending on categories of kidney function.** **A)** all-cause mortality for patients with diabetes mellitus, **B)** cardiovascular mortality for patients with diabetes mellitus, **C)** all-cause mortality for patients without diabetes mellitus, and **D)** cardiovascular mortality for patients without diabetes mellitus according to the three groups of kidney function (depending on the estimated glomerular filtration rate squares: eGFR < 60 mL/min/1.73 m^2^, circles: eGFR 60-89 mL/min/1.73 m^2^, triangles: eGFR: ≥ 90 mL/min/1.73 m^2^). Green shows a protective, yellow a neutral, and red a harmful risk of all-cause and cardiovascular death, respectively. **Abbreviations:** eGFR, estimated glomerular filtration rate; HR, hazard ratios. All tests were two-sided; a p-value < 0.05 was considered statistically significant.

**Supplementary Table S1:** Comparison of eGFR categories with each other

| **Mineral** | **Group comparison** | | | **Mineral** | **Group comparison** | | |
| --- | --- | --- | --- | --- | --- | --- | --- |
|  | **p-Value^b^** | **p-Value^a^** | **Significance** |  | **p-Value^b^** | **p-Value^a^** | **Significance** |
| **Sodium** |  | 0.002 |  | **Iron** |  | < 0.001 |  |
| eGFR < 60 vs. eGFR 60-89 | 0.021 |  | * | eGFR < 60 vs. eGFR 60-89 | < 0.001 |  | *** |
| eGFR ≥ 90 vs. eGFR < 60 | 0.855 |  |  | eGFR ≥ 90 vs. eGFR < 60 | < 0.001 |  | *** |
| eGFR 60-89 vs. eGFR ≥ 90 | 0.009 |  | ** | eGFR 60-89 vs. eGFR ≥ 90 | < 0.001 |  | *** |
| **Potassium** |  | < 0.001 |  | **Zinc** |  | < 0.001 |  |
| eGFR < 60 vs. eGFR 60-89 | < 0.001 |  | *** | eGFR < 60 vs. eGFR 60-89 | 0.001 |  | *** |
| eGFR ≥ 90 vs. eGFR < 60 | < 0.001 |  | *** | eGFR ≥ 90 vs. eGFR < 60 | < 0.001 |  | *** |
| eGFR 60-89 vs. eGFR ≥ 90 | 0.492 |  |  | eGFR 60-89 vs. eGFR ≥ 90 | 0.003 |  | ** |
| **Magnesium** |  | < 0.001 |  | **Copper** |  | < 0.001 |  |
| eGFR < 60 vs. eGFR 60-89 | < 0.001 |  | *** | eGFR < 60 vs. eGFR 60-89 | < 0.001 |  | *** |
| eGFR ≥ 90 vs. eGFR < 60 | < 0.001 |  | *** | eGFR ≥ 90 vs. eGFR < 60 | < 0.001 |  | *** |
| eGFR 60-89 vs. eGFR ≥ 90 | 0.041 |  | * | eGFR 60-89 vs. eGFR ≥ 90 | 0.0233 |  | ** |
| **Calcium** |  | 0.595 |  | **Inorganic phosphate** |  | < 0.001 |  |
| eGFR < 60 vs. eGFR 60-89 | 0.996 |  |  | eGFR < 60 vs. eGFR 60-89 | < 0.001 |  | *** |
| eGFR ≥ 90 vs. eGFR < 60 | 0.743 |  |  | eGFR ≥ 90 vs. eGFR < 60 | < 0.001 |  | *** |
| eGFR 60-89 vs. eGFR ≥ 90 | 0.615 |  |  | eGFR 60-89 vs. eGFR ≥ 90 | 0.303 |  | ** |

^a^ p-Value after post-hoc Tukey test.

^b^ p-Value after family-wise comparison; asterisks [*] indicate significant relationship between eGFR groups (*, p < 0.05; **, p < 0.01; ***, p < 0.001).

**Abbreviation:** eGFR, estimated glomerular filtration rate.

**Supplementary Table S2**: Models of hazard ratios (95% CI) and interaction analyses of minerals and inorganic phosphate according to the three eGFR categories for cardiovascular mortality.

| **Mineral** | **eGFR < 60**^1^ | **eGFR 60-89**^1^ | **eGFR ≥ 90**^1^ | **Interaction** | **For eGFR total** | **p-Value** |
| --- | --- | --- | --- | --- | --- | --- |
| **Sodium** | **-** | **-** | **-** | **-** | 0.90 (0.84 - 0.98) | 0.01 |
| Model 1 | 1.27 (0.77 - 2.11) | 1.10 (0.82 - 1.46) | 1.00 (ref) | - | - | - |
| Model 2 | 1.00 (0.86 - 1.16) | 0.86 (0.77 - 0.96) | 1.00 (ref) | - | - | - |
| Model 3 | 5.67 (4.50 - 7.14) | 2.16 (1.75 - 2.66) | 1.00 (ref) | - | - | - |
| Model 4 | - | - | - | < 0.001 | - | - |
| Model 5 | - | - | - | < 0.001 | - | - |
| Model 6 | - | - | - | < 0.001 | - | - |
| **Potassium** |  |  |  |  | 0.98 (0.91 - 1.06) | 0.596 |
| Model 1 | 1.27 (0.77 - 2.11) | 1.10 (0.82 - 1.46) | 1.00 (ref) | - | - | - |
| Model 2 | 1.03 (0.89 - 1.16) | 0.94 (0.84 - 1.05) | 1.00 (ref) | - | - | - |
| Model 3 | 5.72 (4.54 - 7.20) | 2.13 (1.73 - 2.62) | 1.00 (ref) | - | - | - |
| Model 4 | - | - | - | < 0.001 | - | - |
| Model 5 | - | - | - | < 0.001 | - | - |
| Model 6 | - | - | - | < 0.001 | - | - |
| **Magnesium** |  |  |  |  | 1.01 (0.93 - 1.09) | 0.885 |
| Model 1 | 1.27 (0.77 - 2.11) | 1.10 (0.82 - 1.46) | 1.00 (ref) | - | - | - |
| Model 2 | 1.20 (1.05 - 1.37) | 0.90 (0.81 - 1.01) | 1.00 (ref) | - | - | - |
| Model 3 | 5.68 (4.50 - 7.17) | 2.13 (1.73 - 2.64) | 1.00 (ref) | - | - | - |
| Model 4 | - | - | - | < 0.001 | - | - |
| Model 5 | - | - | - | < 0.001 | - | - |
| Model 6 | - | - | - | < 0.001 | - | - |
| **Calcium** |  |  |  |  | 0.97 (0.90 - 1.05) | 0.017 |
| Model 1 | 1.27 (0.77 - 2.11) | 1.10 (0.82 - 1.46) | 1.00 (ref) | - | - | - |
| Model 2 | 1.01 (0.87 - 1.17) | 0.90 (0.81 - 1.01) | 1.00 (ref) | - | - | - |
| Model 3 | 5.70 (4.53 - 7.18) | 2.13 (1.73 - 2.63) | 1.00 (ref) | - | - | - |
| Model 4 | - | - | - | < 0.001 | - | - |
| Model 5 | - | - | - | < 0.001 | - | - |
| Model 6 | - | - | - | < 0.001 | - | - |
| **Iron** |  |  |  |  | 0.90 (0.83 - 0.98) | < 0.001 |
| Model 1 | 1.27 (0.77 - 2.11) | 1.10 (0.82 - 1.46) | 1.00 (ref) | - | - | - |
| Model 2 | 0.83 (0.71 - 0.96) | 0.76 (0.67 - 0.86) | 1.00 (ref) | - | - | - |
| Model 3 | 5.18 (4.11 - 6.54) | 2.08 (1.68 - 2.56) | 1.00 (ref) | - | - | - |
| Model 4 | - | - | - | < 0.001 | - | - |
| Model 5 | - | - | - | < 0.001 | - | - |
| Model 6 | - | - | - | < 0.001 | - | - |
| **Zinc** |  |  |  |  | 0.79 (0.73 - 0.87) | <0.001 |
| Model 1 | 1.27 (0.77 - 2.11) | 1.10 (0.82 - 1.46) | 1.00 (ref) | - | - | - |
| Model 2 | 0.83 (0.71 - 0.96) | 0.76 (0.67 - 0.86) | 1.00 (ref) | - | - | - |
| Model 3 | 5.18 (4.11 - 6.54) | 2.08 (1.68 - 2.56) | 1.00 (ref) | - | - | - |
| Model 4 | - | - | - | < 0.001 | - | - |
| Model 5 | - | - | - | < 0.001 | - | - |
| Model 6 | - | - | - | < 0.001 | - | - |
| **Copper** |  |  |  |  | 1.19 (1.12 - 1.26) | < 0.001 |
| Model 1 | 1.27 (0.77 - 2.11) | 1.10 (0.82 - 1.46) | 1.00 (ref) | - | - | - |
| Model 2 | 1.15 (1.02 - 1.29) | 1.23 (1.12 - 1.34) | 1.00 (ref) | - | - | - |
| Model 3 | 5.25 (4.16 - 6.63) | 2.09 (1.70 - 2.58) | 1.00 (ref) | - | - | - |
| Model 4 | - | - | - | < 0.001 | - | - |
| Model 5 | - | - | - | 0.017 | - | - |
| Model 6 | - | - | - | 0.004 | - | - |
| **Inorganic Phosphate** | |  |  |  | 1.01 (0.94 - 1.09) | 0.702 |
| Model 1 | 1.27 (0.77 - 2.11) | 1.10 (0.82 - 1.46) | 1.00 (ref) | - | - | - |
| Model 2 | 1.01 (0.87 - 1.16) | 1.04 (0.93 - 1.16) | 1.00 (ref) | - | - | - |
| Model 3 | 5.71 (4.52 – 7.20) | 2.15 (1.74 - 2.65) | 1.00 (ref) | - | - | - |
| Model 4 | - | - | - | < 0.001 | - | - |
| Model 5 | - | - | - | < 0.001 | - | - |
| Model 6 | - | - | - | < 0.001 | - | - |

**Model 1**: eGFR continuous; **Model 2**: Mineral continuous; **Model 3:** Mineral continuous + eGFR continuous; **Model 4:** M + interaction; **Model 5:** eGFR continuous + interaction; **Model 6:** Mineral continuous + eGFR continuous + interaction. **Abbreviations:** eGFR, estimated glomerular filtration rate; HR, Hazard ratio. ^1^ eGFR categories in mL/min/1.73 m^2^. All tests were two-sided; a p-value < 0.05 was considered statistically significant.

**Supplementary Table S3**: Optimal serum concentrations of minerals and inorganic phosphate according to eGFR categories [43].

| **Variable** | **Poor kidney function** | **Satisfactory kidney function** | **Good kidney function** |
| --- | --- | --- | --- |
| **eGFR categories** | **eGFR < 60**^1^ | **eGFR 60-89**^1^ | **eGFR ≥ 90**^1^ |
| **All-cause mortality** |  |  |  |
| Sodium (mmol/L) | 139 - 144 | 140 - 145 | 140 - 147 |
| Potassium (mmol/L) | < 4.2 | 3.9 - 4.5 | 3.9 - 4.4 |
| Magnesium (mmol/L) | < 0.94 | 0.80 - 1.00 | 0.80 - 1.04 |
| Calcium (mmol/L) | 2.26 - 2.57 | > 2.30 | > 2.30 |
| Iron (µg/dL) | 57 - 125 | 76 - 214 | 72 - 144 |
| Zinc (µmol/L) | > 76 | > 88 | > 83 |
| Copper (µg/dL) | < 110 | < 105 | < 112 |
| Inorganic phosphate (mg/dL) | 3.3 - 4.8 | > 3.8 | 2.9 - 3.8 |
| **Cardiovascular mortality** |  |  |  |
| Sodium (mmol/L) | 138 - 144 | 140  s 145 | > 140 |
| Potassium (mmol/L) | < 4.4 | 3.93 - 4.50 | 3.99 - 4.52 |
| Magnesium (mmol/L) | < 0.90 | 0.80 - 0.97 | 0.78 - 0.96 |
| Calcium (mmol/L) | 2.21 - 2.43 | 2.27-2.50 | > 2.36 |
| Iron (µg/dL) | > 70 | 73 - 161 | 74 - 149 |
| Zinc (µmol/L) | > 78 | > 86 | > 82 |
| Copper (µg/dL) | < 110 | < 102 | < 112 |
| Inorganic phosphate (mg/dL) | 3.3 - 4.6 | < 3.7 | 3.0 - 4.0 |

Optimal concentration ranges after determining the hazard ratio curves vs. serum concentrations with the intercept of a hazard ratio of 1. **Abbreviation:** eGFR, estimated glomerular filtration rate.

^1^ eGFR categories in mL/min/1.73 m^2^.

All tests were two-sided; a p-value < 0.05 was considered statistically significant.

**Supplementary Table S4:** Study characteristics according to three eGFR categories in individuals with diabetes

| **Variable** | **Total** | **Poor kidney function** | **Satisfactory kidney function** | **Good kidney function** |  |  |
| --- | --- | --- | --- | --- | --- | --- |
| **eGFR categories** |  | **eGFR < 60**^1^ | **eGFR 60-89**^1^ | **eGFR ≥ 90**^1^ | **p**^a^ | **FDR**^b^ |
| **N** | **1,321** | **257** | **696** | **368** | **-** | **-** |
| eGFR (ml/min per 1.73 m^2^) | 76.9 ± 20.5 | 45.6 ± 11.4 | 76.3 ± 8.7 | 100.0 ± 7.6 | < 0.001 | < 0.001 |
| Age (years) | 65.5 ± 0.3 | 69.8 ± 7.8 | 65 ± 7.5 | 58.8 ± 8.6 | < 0.001 | < 0.001 |
| Female sex (%) | 30.2 | 37.3 | 29.1 | 19.7 | < 0.001 | < 0.001 |
| Body mass index (kg/m^2^) | 28.3 ± 0.1 | 28.3 ± 4.6 | 28.3 ± 4.1 | 28.3 ± 4.3 | 0.950 | 0.950 |
| LDL-cholesterol (mg/dL) | 113.8 ± 0.9 | 112.0 ± 34.1 | 116.0 ± 32.1 | 115.0 ± 36.7 | 0.122 | 0.152 |
| HDL-cholesterol (mg/dL) | 36.5 ± 0.3 | 35.9 ± 10.2 | 37.1 ± 9.1 | 36.8 ± 10.5 | 0.113 | 0.147 |
| Apolipoprotein A1 (mg/dL) | 125.1 ± 0.7 | 123.0 ± 23.9 | 127.0 ± 22.8 | 126.0 ± 25.1 | 0.065 | 0.088 |
| Apolipoprotein A2 (mg/dL) | 39.6 ± 0.3 | 37.4 ± 8.8 | 40.4 ± 9.2 | 42.3 ± 10.5 | < 0.001 | < 0.001 |
| Triglycerides (mg/dL) | 159 (120 - 217) | 161 (120 - 217) | 157 (120 - 210) | 160 (120 - 230) | 0.552 | 0.612 |
| Fasting glucose (mg/dL) | 126.0 (105.3 - 153.9) | 124.0 (102.0 - 158.0) | 121.0 (106.0 - 147.0) | 129.0 (108.0 - 157.0) | 0.061 | 0.084 |
| Glycated haemoglobin A_1c_ (%) | 7.2 ± 0.0 | 7.4 ± 1.5 | 7.1 ± 1.3 | 7.2 ± 1.7 | 0.005 | 0.009 |
| Systolic blood pressure (mmHg) | 145.3 ± 0.7 | 147.0 ± 24.5 | 146.0 ± 23.3 | 142.0 ± 23.1 | 0.030 | 0.042 |
| Diastolic blood pressure (mmHg) | 81.4 ± 0.3 | 80.0 ± 11.7 | 82.6 ± 11.7 | 82.2 ± 10.5 | 0.001 | 0.002 |
| High-sensitive C-reactive protein (mg/L) | 4.5 (1.8 - 10.2) | 5.7 (2.3 - 11.2) | 4.1 (1.7 - 9.3) | 3.5 (1.4 - 9.1) | < 0.001 | < 0.001 |
| NT-pro-BNP (pg/mL) | 410 (151 – 1,234) | 800 (305 – 2,180) | 336 (130 - 998) | 201 (68 - 524) | < 0.001 | < 0.001 |
| Galectin-3 (ng/mL) | 15.7 (12.1 - 20.5) | 19.5 (14.9 - 24.8) | 15.1 (11.7 - 18.7) | 13 (10.2 - 15.8) | < 0.001 | < 0.001 |
| Creatinine (mg/dL) | 0.9 (0.8 - 1.1) | 1.1 (1 - 1.3) | 0.9 (0.8 - 1.0) | 0.8 (0.7 - 0.9) | < 0.001 | < 0.001 |
| Renin (pg/mL) | 22.0 (11.0 - 51.0) | 28.0 (13.0 - 73.0) | 21.0 (10.0 - 45.0) | 18.0 (9.0 - 33.0) | < 0.001 | < 0.001 |
| Blood urea nitrogen (mg/dL) | 42.8 ± 0.5 | 52.8 ± 22.4 | 37.3 ± 9.2 | 33.3 ± 8.7 | < 0.001 | < 0.001 |
| Uric acid (mg/dL) | 5.1 (4.2 - 6.3) | 5.7 (4.7 - 7.2) | 4.9 (4.0 - 6.0) | 4.5 (3.8 - 5.5) | < 0.001 | < 0.001 |
| Trimethylamine N-oxide (µmol/L) | 4.66 (3.22 - 6.89) | 5.80 (4.12 - 9.05) | 4.33 (3.24 - 6.06) | 3.47 (2.43 - 4.91) | < 0.001 | < 0.001 |
| Symmetric dimethylamine (µmol/L) | 0.55 (0.46 - 0.68) | 0.66 (0.55 - 0.83) | 0.52 (0.45 - 0.60) | 0.45 (0.40 - 0.52) | < 0.001 | < 0.001 |
| Coronary artery disease (%) | 85.1 | 85.9 | 84.9 | 84 | 0.740 | 0.803 |
| Hypertension (%) | 81.9 | 83.9 | 83.1 | 76.9 | 0.025 | 0.037 |
| Hypertension medication (%) | 91.0 | 96.4 | 88.5 | 86.5 | < 0.001 | < 0.001 |
| Smoking status |  |  |  |  |  |  |
| - Smoking: active/former/never (%) | 21.1 / 44.8 / 34.1 | 14.1 / 47.2 / 38.7 | 20.5 / 46.3 / 33.2 | 33.5 / 38.8 / 27.7 | < 0.001 | < 0.001 |
| - Cigarette packs per day (%) | 20.0 (7.5 - 40.0) | 18.8 (6.0 - 37.5) | 20.0 (7.5 - 40.2) | 21.0 (10.0 - 40.0) | 0.024 | 0.037 |
| Alcohol consumption |  |  |  |  |  |  |
| - Total alcohol consumption (g/d ethanol) | 14.8 ± 0.6 | 11.3 ± 17.6 | 17.5 ± 24.8 | 17.2 ± 26.8 | < 0.001 | < 0.001 |

Values are given as either median (25^th^ and 75^th^ percentile) for non-normally distributed data, as mean ± SD for normally distributed data or percentage for categorial data. ^1^ eGFR categories in mL/min/1.73 m^2^.

^a^ ANOVA (non-normally distributed variables were log transformed before entering analyses) or χ^2^ test.

^b^ p-Value after FDR correction. All tests were two-sided; a p-value < 0.05 was considered statistically significant.

**Abbreviations:** eGFR, estimated glomerular filtration rate; FDR, false detection rate; HDL, high-density lipoprotein; LDL, low-density lipoprotein; NT-pro-BNP, N-terminal pro-B-type natriuretic peptide-1.

**Supplementary Table S5**: Serum concentrations of minerals and inorganic phosphate according to the three eGFR categories in individuals with diabetes mellitus.

| **Variable** | **Total** | **Poor kidney function** | **Satisfactory kidney function** | **Good kidney function** |  |  |
| --- | --- | --- | --- | --- | --- | --- |
| **eGFR categories** |  | **eGFR < 60**^1^ | **eGFR 60-89**^1^ | **eGFR ≥ 90**^1^ | **p**^a^ | **FDR**^b^ |
| **N** | **1,321** | **257** | **696** | **368** | **-** | **-** |
| Sodium (mmol/L) | 141 (139 - 143) | 141 (139 - 143) | 141 (139 - 143) | 141 (139 - 142) | 0.120 | 0.152 |
| Potassium (mmol/L) | 4.2 (4.0 - 4.4) | 4.2 (4.0 - 4.4) | 4.2 (4.0 - 4.3) | 4.2 (4.0 - 4.4) | 0.023 | 0.037 |
| Magnesium (mmol/L) | 0.85 (0.78 - 0.91) | 0.87 (0.81 - 0.93) | 0.84 (0.78 - 0.9) | 0.83 (0.76 - 0.89) | < 0.001 | < 0.001 |
| Calcium (mmol/L) | 2.33 (2.26 - 2.40) | 2.33 (2.26 - 2.41) | 2.33 (2.26 - 2.39) | 2.32 (2.27 - 2.38) | 0.357 | 0.414 |
| Iron (µg/dL) | 85 (63 - 110) | 78 (59 - 104) | 89 (66 - 111) | 89 (70 - 115) | < 0.001 | < 0.001 |
| Zinc (µmol/L) | 86 (77 - 96) | 83 (73 - 92) | 87 (79 - 98) | 90 (80 - 99) | < 0.001 | < 0.001 |
| Copper (µg/dL) | 109 (93 - 127) | 111 (96 - 129) | 108 (93 - 125) | 106 (92 - 126) | 0.012 | 0.019 |
| Inorganic phosphate (mg/dL) | 3.5 (3.1 - 3.9) | 3.7 (3.3 - 4.0) | 3.6 (3.2 - 4.0) | 3.5 (3.1 - 3.9) | 0.003 | 0.005 |

Values are given as median (25^th^ and 75^th^ percentile) for non-normally distributed data, as mean ± SD for normally distributed data, and as percentage for categorial data. ^1^ eGFR categories in mL/min/1.73 m^2^.

^a^ ANOVA (non-normally distributed variables were log transformed before entering analyses) or χ^2^ test.

^b^ p-Value after FDR correction. All tests were two-sided; a p-value < 0.05 was considered statistically significant.

**Abbreviations:** eGFR, estimated glomerular filtration rate; FDR, false detection rate.

**Supplementary Table S6:** Study characteristics according to three eGFR categories in individuals without diabetes mellitus.

| **Variable** | **Total** | **Poor kidney function** | **Satisfactory kidney function** | **Good kidney function** |  |  |
| --- | --- | --- | --- | --- | --- | --- |
| **eGFR categories** | **eGFR <60** | **eGFR < 60**^1^ | **eGFR 60-89**^1^ | **eGFR ≥ 90**^1^ | **P**^a^ | **FDR**^b^ |
| **N** | **1,986** | **199** | **946** | **841** | **-** | **-** |
| eGFR (ml/min per 1.73 m^2^) | 84.9 ± 19.2 | 46.9 ± 11.7 | 77.6 ± 8.3 | 102.1 ± 8.1 | < 0.001 | < 0.001 |
| Age (years) | 60.8 ± 0.3 | 68.6 ± 8.5 | 62.8 ± 8.7 | 53.6 ± 10.3 | < 0.001 | < 0.001 |
| Female sex (%) | 30.5 | 41.3 | 31.8 | 21.6 | < 0.001 | < 0.001 |
| Body mass index (kg/m^2^) | 27.0 ± 0.1 | 27.1 ± 3.8 | 27.0 ± 3.8 | 26.8 ± 3.8 | 0.215 | 0.259 |
| LDL-cholesterol (mg/dL) | 118.4 ± 0.8 | 116.0 ± 34.4 | 119.0 ± 33.6 | 119.0 ± 34.9 | 0.265 | 0.287 |
| HDL-cholesterol (mg/dL) | 40.2 ± 0.3 | 39.6 ± 11.7 | 40.9 ± 11.0 | 40.1 ± 10.8 | 0.120 | 0.152 |
| Apolipoprotein A1 (mg/dL) | 132.3 ± 0.6 | 131.0 ± 26.0 | 134.0 ± 25.0 | 132.0 ± 25.1 | 0.095 | 0.123 |
| Apolipoprotein A2 (mg/dL) | 42.9 ± 0.2 | 39.9 ± 8.7 | 43.2 ± 8.9 | 44.7 ± 9.2 | < 0.001 | < 0.001 |
| Triglycerides (mg/dL) | 137 (103 - 189) | 139 (110 - 188) | 132 (97 - 189) | 140 (103 - 191) | 0.234 | 0.276 |
| Fasting glucose (mg/dL) | 97.4 (91.1 - 104.2) | 98.3 (91.0 - 105.0) | 97.4 (91.4 - 104.0) | 97.2 (91.1 - 104.0) | 0.265 | 0.287 |
| Glycated haemoglobin A_1c_ (%) | 5.7 ± 0.0 | 5.8 ± 0.4 | 5.7 ± 0.4 | 5.6 ± 0.5 | < 0.001 | < 0.001 |
| Systolic blood pressure (mmHg) | 138.4 ± 0.5 | 143.0 ± 24.7 | 140.0 ± 23.2 | 134.0 ± 20.8 | < 0.001 | < 0.001 |
| Diastolic blood pressure (mmHg) | 80.7 ± 0.3 | 81.0 ± 12.2 | 81.6 ± 11.2 | 79.7 ± 11.0 | 0.004 | 0.006 |
| High-sensitive C-reactive protein (mg/L) | 2.8 (1.1 - 7.0) | 4.3 (1.7 - 9.6) | 2.5 (1.1 - 6.0) | 2.1 (0.9 - 6.0) | < 0.001 | < 0.001 |
| NT-pro-BNP (pg/mL) | 233 (93 - 636) | 570 (245 - 1690) | 231 (99 - 575) | 128 (58 - 323) | < 0.001 | < 0.001 |
| Galectin-3 (ng/mL) | 13.9 (10.5 - 18.0) | 18 (14.2 - 22.7) | 13.5 (10.5 - 17.2) | 12 (9.2 - 14.9) | < 0.001 | < 0.001 |
| Creatinine (mg/dL) | 0.9 (0.8 - 1.0) | 1.1 (0.9 - 1.2) | 0.9 (0.8 - 1.0) | 0.8 (0.8 - 0.9) | < 0.001 | < 0.001 |
| Renin (pg/mL) | 17 (9 - 36) | 20 (10 - 49) | 15 (8 - 34) | 18 (10 - 34) | < 0.001 | < 0.001 |
| Blood urea nitrogen (mg/dL) | 37.14 ± 0.28 | 45.9 ± 17 | 36.3 ± 8.63 | 31.6 ± 7.58 | < 0.001 | < 0.001 |
| Uric acid (mg/dL) | 4.7 (3.8 - 5.7) | 5.2 (4.2 - 6.7) | 4.6 (3.8 - 5.5) | 4.4 (3.6 - 5.3) | < 0.001 | < 0.001 |
| Trimethyl-N aminoxide (µmol/L) | 4.0 (2.83 - 5.65) | 5.23 (3.71 - 7.48) | 4.03 (3.04 - 5.41) | 3.26 (2.38 - 4.61) | < 0.001 | < 0.001 |
| Symmetric dimethylamine (µmol/L) | 0.53 (0.45 - 0.63) | 0.66 (0.56 - 0.81) | 0.53 (0.46 - 0.616) | 0.47 (0.41 - 0.54) | < 0.001 | < 0.001 |
| Coronary artery disease (%) | 73.1 | 79.6 | 73.6 | 68.0 | < 0.001 | < 0.001 |
| Hypertension (%) | 66.6 | 78.5 | 71.1 | 54.4 | < 0.001 | < 0.001 |
| Hypertension medication (%) | 84.0 | 92.5 | 83.5 | 78.0 | < 0.001 | < 0.001 |
| Smoking status |  |  |  |  |  |  |
| - Smoking: active/former/never (%) | 24.9 / 38.6 / 36.5 | 15.7 / 39.0 / 45.4 | 22.6 / 38.3 / 39.1 | 33.3 / 38.6 / 28.1 | < 0.001 | < 0.001 |
| - Cigarette packs per day (%) | 15.0 (5.0 - 33.4) | 12.0 (3.6 – 30.0) | 15.0 (5.0 - 32.5) | 18.0 (6.0 - 36.8) | < 0.001 | 0.001 |
| Alcohol consumption |  |  |  |  |  |  |
| - Total alcohol consumption (g/d ethanol) | 16.7 ± 0.6 | 14.5 ± 21.0 | 16.6 ± 23.5 | 18.3 ± 26.8 | 0.022 | 0.031 |

Values are given as either median (25^th^ and 75^th^ percentile) for non-normally distributed data, as mean ± SD for normally distributed data or percentage for categorial data. ^1^ eGFR categories in mL/min/1.73 m^2^.

^a^ANOVA (non-normally distributed variables were log transformed before entering analyses) or χ^2^ test.

^b^p-Value after FDR correction. All tests were two-sided; a p-value < 0.05 was considered statistically significant.

**Abbreviations:** eGFR, estimated glomerular filtration rate; FDR, false detection rate; HDL, high-density lipoprotein; LDL, low-density lipoprotein; NT-pro-BNP, N-terminal pro-B- type natriuretic peptide-1.

**Supplementary Table S7:** Serum concentrations of minerals and inorganic phosphate according to three eGFR categories in individuals without diabetes mellitus.

| **Variable** | **Total** | **Poor kidney function** | **Satisfactory kidney function** | **Good kidney function** |  |  |
| --- | --- | --- | --- | --- | --- | --- |
| **eGFR categories** |  | **eGFR < 60**^1^ | **eGFR 60-89**^1^ | **eGFR ≥ 90**^1^ | **p**^a^ | **FDR**^b^ |
| **N** | **1,986** | **199** | **946** | **841** | **-** | **-** |
| Sodium (mmol/L) | 140 (140 - 143) | 142 (140 - 143) | 142 (140 - 143) | 141 (140 - 143) | 0.074 | 0.101 |
| Potassium (mmol/L) | 4.2 (4.0 - 4.4) | 4.2 (4.0 - 4.4) | 4.2 (4.0 - 4.3) | 4.2 (4.0 - 4.4) | 0.261 | 0.287 |
| Magnesium (mmol/L) | 0.85 (0.80 - 0.91) | 0.86 (0.81 - 0.93) | 0.85 (0.79 - 0.91) | 0.84 (0.79 - 0.91) | 0.002 | 0.003 |
| Calcium (mmol/L) | 2.33 (2.26 - 2.39) | 2.33 (2.27 - 2.41) | 2.31 (2.25 - 2.38) | 2.33 (2.27 - 2.39) | 0.076 | 0.101 |
| Iron (µg/dL) | 91 (69 - 116) | 86 (63 - 110) | 93 (70 - 119) | 94 (72 - 120) | < 0.001 | < 0.001 |
| Zinc (µmol/L) | 87 (77 - 96) | 84 (75 - 93) | 87 (77 - 95) | 88 (79 - 97) | < 0.001 | < 0.001 |
| Copper (µg/dL) | 103 (91 - 120) | 108 (94 - 124) | 102 (90 - 118) | 101 (90 - 118) | < 0.001 | < 0.001 |
| Inorganic phosphate (mg/dL) | 3.5 (3.1 - 3.9) | 3.6 (3.2 - 4.0) | 3.5 (3.1 - 3.8) | 3.5 (3.1 - 3.8) | < 0.001 | < 0.001 |

Values are given as median (25^th^ and 75^th^ percentile), for non-normally distributed data, as mean ± SD for normally distributed data, and as percentage for categorial data. ^1^ eGFR categories in mL/min/1.73 m^2^.

^a^ ANOVA (non-normally distributed variables were log transformed before entering analyses) or χ^2^ test.

^b^ p-Value after FDR correction. All tests were two-sided; a p-value < 0.05 was considered statistically significant.

**Abbreviations:** eGFR, estimated glomerular filtration rate*;* FDR, false detection rate*.*
